# Three new species of the subgenus *Neoribates (Neoribates)* (Acari, Oribatida, Parakalummidae) from Nepal

**DOI:** 10.3897/zookeys.431.8120

**Published:** 2014-08-05

**Authors:** Sergey G. Ermilov, Jochen Martens

**Affiliations:** 1 Tyumen State University, Tyumen, Russia; 2 Johannes Gutenberg University, Mainz, Germany

**Keywords:** Oribatid mites, new species, *Neoribates (Neoribates)*, Nepal

## Abstract

Three new parakalummid mites of the subgenus *Neoribates (Neoribates)*, *N. (N.) parabulanovae* **sp. n.**, *N. (N.) paramacrosacculatus* **sp. n.** and *N. (N.) pararotundus* **sp. n.**, are described from Nepalese soils. *Neoribates (Neoribates) parabulanovae* **sp. n.** is morphologically most similar to *N. (N.) bulanovae* Grishina, 2009, *N. (N.) rotundus* Aoki, 1982 and *N. (N.) setiger* Balogh & Mahunka, 1978, however, it differs from *N. (N.) bulanovae* by the body length, body and leg integument, morphology of bothridial setae, absence of aggenital setae, length of interlamellar setae and location of adanal setae *ad*_3_; from *N. (N.) rotundus* by the body size, body integument, morphology of bothridial setae and length of interlamellar setae; from *N. (N.) setiger* by the body size, number of genital setae and absence of aggenital setae. *Neoribates (Neoribates) paramacrosacculatus* **sp. n.** is morphologically most similar to *N. (N.) macrosacculatus* Aoki, 1966, however, it differs from the latter by the body size, body integument, length and morphology of bothridial setae, number of genital setae, absence of lamellar setae and length of interlamellar setae. *Neoribates (Neoribates) pararotundus* **sp. n.** is morphologically most similar to *N. (N.) rotundus*, however, it differs from the latter by the number of notogastral setal alveoli, body integument and length of interlamellar setae.

## Introduction

During a taxonomic survey of Nepalese oribatid mite fauna^1^ (Acari, Oribatida) we found three new species of the genus *Neoribates* Berlese, 1914 (subgenus *Neoribates (Neoribates)* Berlese, 1914). The main goal of this paper is to describe these species. Earlier the only one species of *Neoribates* has been found in Nepal (Ermilov and Martens 2014): *Neoribates (Neoribates) aurantiacus* (Oudemans, 1914).

*Neoribates (Neoribates)* has a cosmopolitan distribution except the Ethiopian and Antarctic regions, comprises about 40 species (Subías 2004, updated 2014). The main generic characters of this subgenus are summarized by Ermilov and Kalúz (2013). The identification keys to some species of *Neoribates (Neoribates)* were provided earlier (Balogh and Balogh 2002; Grishina and Vladimirova 2009; Ermilov and Kalúz 2013).

## Material and methods

The collection locality and habitat of the new species are given in the respective "Material examined" sections.

Holotypes and paratypes were mounted in lactic acid on temporary cavity slides for measurement and illustration. The body length was measured in lateral view, from the tip of the rostrum to the posterior edge of the ventral fig. The notogastral width refers to the maximum width in dorsal aspect. Lengths of body setae were measured in lateral aspect. All body measurements are presented in micrometers. Formula for leg setation is given in parentheses according to the sequence trochanter–femur–genu–tibia–tarsus (famulus included). Formula for leg solenidia is given in square brackets according to the sequence genu–tibia–tarsus. General terminology used in this paper follows that of Norton and Behan-Pelletier (2009).

## Taxonomy

### Neoribates (Neoribates) parabulanovae sp. n.

Taxon classificationAnimaliaSarcoptiformes Parakalummidae

http://zoobank.org/D38AE3B5-F33D-4CA8-8CCA-2836F9EE1C00

Figs 1 –4

#### Diagnosis.

Body size: 946–1062 × 697–763. Body surface densely microfoveolate; anterior part of rostrum, subcapitular genae and leg segments III, IV with larger, sparse foveoles. Rostral, lamellar and interlamellar setae well developed, barbed. Bothridial setae setiform, slightly barbed. Saccular openings short. Subcapitular setae *h* and *m* longer than *a*; *h* thickest. Five pairs of genital setae present. Aggenital setae absent. Adanal setae short, *ad*_3_ inserted in preanal position. Tridactylous.

#### Description.

*Measurements*. Body length: 1012 (holotype), 946–1062 (four paratypes); notogaster width: 697 (holotype), 713–763 (four paratypes).

*Integument*. Body color brown. Body surface densely microfoveolate (diameter of microfoveoles less than 1; visible only under high magnification in dissected specimens, × 1000). Anterior part of rostrum, subcapitular genae and leg segments III, IV with larger, sparse foveoles (their diameter up to 4).

*Prodorsum*. Rostrum weakly protruding, rounded. A small tubercle (*tb*) is present in medial part nearly to rostrum. Lamellae distinct, reaching the insertions of alveoli of lamellar setae. Rostral setae (*ro*, 118–131) setiform, barbed. Lamellar (*le*, 192–200) and interlamellar (*in*, 278–287) setae setiform, with short attenuate tip, barbed. Exobothridial setae short (8), thin, smooth. Bothridial setae (*ss*, 192–200) setiform, slightly barbed on dorsal side.

*Notogaster*. Anterior notogastral margin convex. Notogastral setae represented by 10 pairs of alveoli or microsetae (1). Four pairs of sacculi (*Sa*, *S1*–*S3*) present, their openings short, thin. Alveoli/microsetae *lp* inserted laterally to *S1*. Opisthonotal gland openings (*gla*) located laterally to sacculi *S1*. Lyrifissures *im* located between *gla* and setal alveolus *lm.*

*Gnathosoma*. Generally, morphology of subcapitulum, palps and chelicerae typical as for most *Neoribates (Neoribates)* (see for example Grishina and Vladimirova 2009; Ermilov and Kalúz 2013; Ermilov and Anichkin 2014). Subcapitulum longer than wide (188–196 × 143–147). Subcapitular setae setiform, slightly barbed; *h* (53–57) thicker than *a* (28–32), *m* (53–57) thinnest. Two pairs of adoral setae (20) setiform, densely bilaterally barbed. Palps (length 98) with setation 0–2–1–3–9(+ω). Solenidion attached to eupathidium. Chelicerae (length 188–196) with two barbed setae; *cha* (61) longer than *chb* (49). Trägårdh’s organ distinct.

*Epimeral and lateral podosomal regions*. Epimeral setal formula: 3–1–3–3. All setae setiform, slightly barbed; *1a*, *1c*, *2a*, *3a* (32–36) shorter than *1b*, *3b*, *4a*, *4b* (61–65) and *3c*, *4c* (77–86). Setae *1a*, *2a*, *3a* located close to each other. Pedotecta I, II, discidia and circumpedal carinae (*cp*) normally developed.

*Anogenital region*. Five pairs of genital (*g*_1_–*g*_5_, 32–36), three pairs of adanal (*ad*_1_–*ad*_3_, 24–32) and two pairs of anal (*an*_1_, *an*_2_, 24–32) setae setiform, with sparse barbs. Genital setae *g*_1_ and *g*_2_ little thicker than *g*_3_–*g*_5_. Aggenital setae and their alveoli absent. Lyrifissures *iad* located in paraanal position, usually weakly diagonally to the anal aperture. Adanal setae *ad*_3_ inserted in preanal position.

*Legs*. Generally, morphology of leg segments, setae and solenidia typical for *Neoribates (Neoribates)* (see for example: Travé 1972; Grishina and Vladimirova 2009; Ermilov and Anichkin 2014). Leg tarsi with three smooth claws. Formulae of leg setation and solenidia: I (1–5–3–4–20) [1–2–2], II (1–5–3–4–15) [1–1–2], III (2–3–1–3–15) [1–1–0], IV (1–2–2–4–12) [0–0–0]; homology of setae and solenidia indicated in Table 1. Famulus (ε) short, straight, weakly dilated distally, blunted, inserted between ω_2_ and *ft*’’. Many setae well barbed. Solenidia ω_2_ on tarsi I, ω_1_, ω_2_ on tarsi II and σ on genua III thickened, blunted; other solenidia thinner, with attenuate tip.

#### Material examined.

Five specimens (holotype: male; four paratypes: three males, one female) of *Neoribates (Neoribates) parabulanovae* sp. n. are from central Nepal, 28°34'N, 83°98'E, 2100 m a.s.l., Kaski District, above Dhumpus, broadleaved forest, soil, 08–10.V.1980, collected by J. Martens & A. Ausobsky.

#### Type deposition.

The holotype and one paratype are deposited in the collection of the Senckenberg Institution Frankfurt, Germany; three paratypes are deposited in the collection of the Tyumen State University Museum of Zoology, Tyumen, Russia.

#### Etymology.

The prefix *para* is Latin meaning “near” and refers to the similarity between the new species and the species *Neoribates (Neoribates) bulanovae* Grishina, 2009.

#### Remarks.

*Neoribates (Neoribates) parabulanovae* sp. n. is morphologically similar to *Neoribates (Neoribates) bulanovae* Grishina, 2009 (see Grishina and Vladimirova 2009) from eastern Mediterranean, *Neoribates (Neoribates) rotundus* Aoki, 1982 from Japan and Taiwan and *Neoribates (Neoribates) setiger* Balogh & Mahunka, 1978 from Australia in having the setiform bothridial setae. However, the new species differs from *Neoribates (Neoribates) bulanovae* by the larger body length (946–1062 versus 830–904 in *Neoribates (Neoribates) bulanovae*), foveolate body integument and leg segments III, IV (versus smooth in *Neoribates (Neoribates) bulanovae*), slightly barbed bothridial setae (versus well barbed in *Neoribates (Neoribates) bulanovae*), absence of aggenital setae (versus present in *Neoribates (Neoribates) bulanovae*), interlamellar setae longer than bothridial setae (versus similar in length in *Neoribates (Neoribates) bulanovae*) and adanal setae *ad*_3_ located anteriorly to adanal lyrifissures (versus laterally in *Neoribates (Neoribates) bulanovae*); from *Neoribates (Neoribates) rotundus* by the larger body size (946–1062 × 697–763 versus 810 × 660 in *Neoribates (Neoribates) rotundus*), foveolate body integument (versus smooth in *Neoribates (Neoribates) rotundus*), slightly barbed bothridial setae (versus smooth in *Neoribates (Neoribates) rotundus*) and interlamellar setae longer than bothridial setae (versus shorter in *Neoribates (Neoribates) rotundus*); from *Neoribates (Neoribates) setiger* by the larger body size (946–1062 × 697–763 versus 559–583 × 389–450 in *Neoribates (Neoribates) setiger*), presence of five pairs of genital setae (versus four pairs in *Neoribates (Neoribates) setiger*) and absence of aggenital setae (versus present in *Neoribates (Neoribates) setiger*).

**Figures 1–2.**
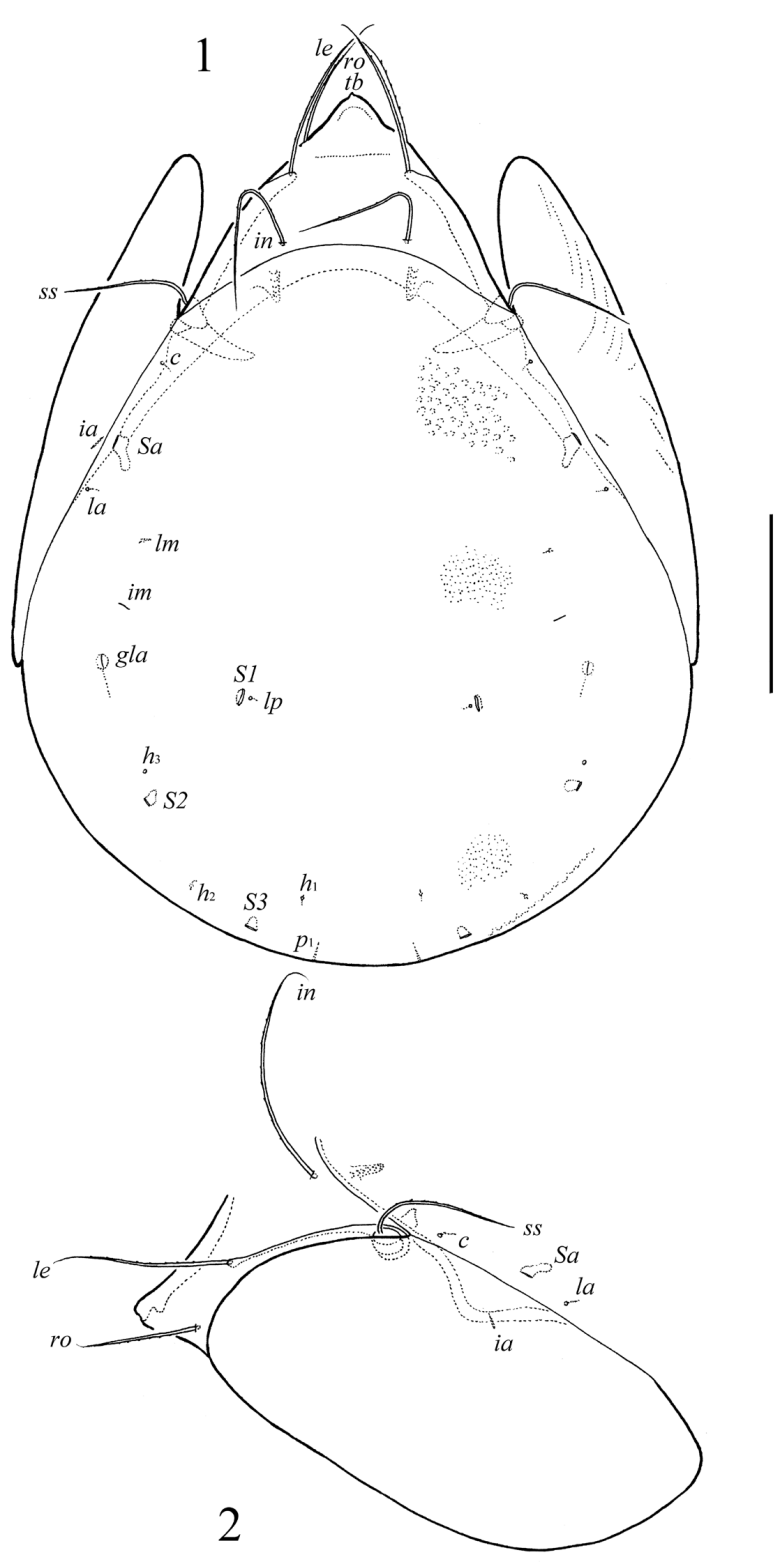
*Neoribates (Neoribates) parabulanovae* sp. n., adult: **1** dorsal view **2** lateral view of prodorsum, anterior part of notogaster, and pteromorph. Scale bar 200 μm.

**Figures 3–4.**
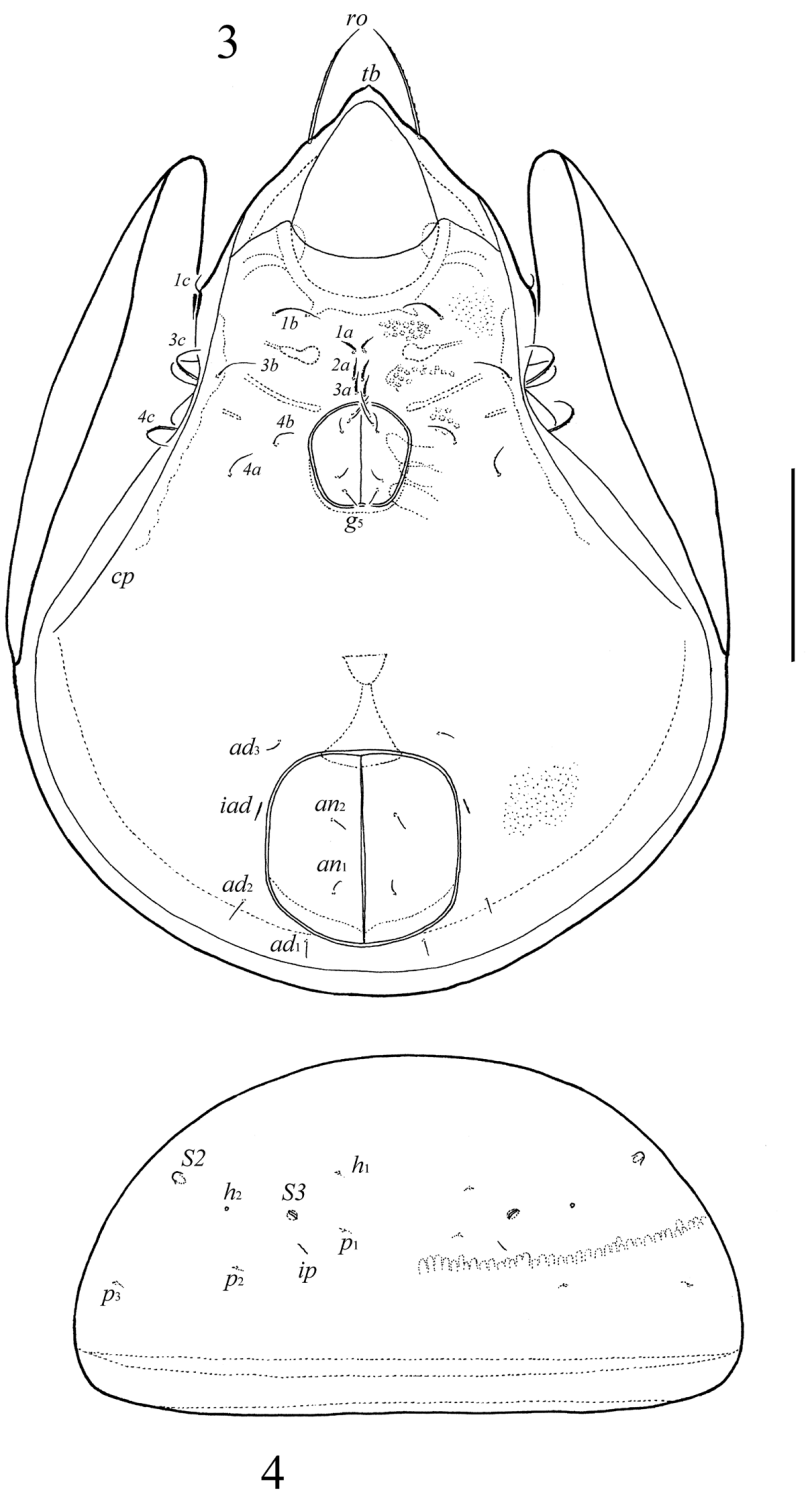
*Neoribates (Neoribates) parabulanovae* sp. n., adult: **3** ventral view (gnathosoma and legs not illustrated) **4** posterior view of notogaster (pteromorphs not illustrated). Scale bar 200 μm.

**Table 1.** Leg setation and solenidia of adult *Neoribates (Neoribates) parabulanovae* sp. n. (same data for *Neoribates (Neoribates) paramacrosacculatus* sp. n. and *Neoribates (Neoribates) pararotundus* sp. n.)

**Leg**	**Trochanter**	**Femur**	**Genu**	**Tibia**	**Tarsus**
I	*v*'	*d, (l), bv'', v*''	*(l), v*', σ	*(l), (v)*, φ_1_, φ_2_	*(ft), (tc), (it), (p), (u), (a), s, (pv), v', (pl), l*'', ε, ω_1_, ω_2_
II	*v*'	*d, (l), bv'', v*''	*(l), v*', σ	*(l), (v)*, φ	*(ft), (tc), (it), (p), (u), (a), s, (pv)*, ω_1_, ω_2_
III	*l', v*'	*d, l', ev*'	*l*', σ	*l', (v)*, φ	*(ft), (tc), (it), (p), (u), (a), s, (pv)*
IV	*v*'	*d, ev*'	*d, l*'	*d, l', (v)*	*ft'', (tc), (p), (u), (a), s, (pv)*

Roman letters refer to normal setae (*e* to famulus), Greek letters to solenidia. Single prime (') marks setae on anterior and double prime (") setae on posterior side of the given leg segment. Parentheses refer to a pair of setae.

### Neoribates (Neoribates) paramacrosacculatus sp. n.

Taxon classificationAnimaliaSarcoptiformes Parakalummidae

http://zoobank.org/EBD29A23-2777-4E3B-9044-61D895BB09F4

Figs 5 –8

#### Diagnosis.

Body size: 780–830 × 547–581. Body surface densely microfoveolate. Rostral setae of medium size setiform. Lamellar setae represented by alveoli. Interlamellar setae represented by microsetae. Bothridial setae lanceolate, barbed; stalk and elongate head similar in length. Saccular openings long. Subcapitular setae *m* longer than *a* and *h*; *a* thickest. Five pairs of genital setae present. Medial parts of anal figs striate. Adanal setae *ad*_3_ inserted in lateral position. Tridactylous.

#### Description.

*Measurements*. Body length: 780 (holotype), 780, 830 (two paratypes); notogaster width: 547 (holotype), 581 (both paratypes).

*Integument*. Body color brown. Body surface densely microfoveolate (diameter of microfoveoles less than 1; visible only under high magnification in dissected specimens, × 1000). Medial parts of anal figs with longitudinal stria.

*Prodorsum*. Rostrum weakly protruding, rounded. Lamellae distinct, reaching the insertions of alveoli of lamellar setae. Rostral setae (53) setiform, slightly barbed, present in one paratype and brokened (only basal parts visible) in holotype and one paratype. Lamellar setae represented by alveoli. Interlamellar setae represented by microsetae (2). Exobothridial setae short (12), thin, smooth. Bothridial setae (77–82) of medium size, lanceolate, barbed; stalk and elongate head similar in length.

*Notogaster*. Anterior notogastral margin convex. Notogastral setae represented by 10 pairs of alveoli or microsetae (2–4). Four pairs of sacculi present, their openings long, thin, slit-like. Alveoli/microsetae *lp* inserted laterally to *S1*. Opisthonotal gland openings located laterally to succuli *S1*. Lyrifissures *im* located between *gla* and setal alveolus *lm.*

*Gnathosoma*. Generally, morphology of subcapitulum, palps and chelicerae typical as for most *Neoribates (Neoribates)* (see for example Grishina and Vladimirova 2009; Ermilov and Kalúz 2013; Ermilov and Anichkin 2014). Subcapitulum longer than wide (159–164 × 127–131). Subcapitular setae setiform, slightly barbed; *a* (16) thicker than *m* (24) and *h* (12). Two pairs of adoral setae (16) setiform, densely bilaterally barbed. Palps (length 110) with setation 0–2–1–3–9(+ω). Solenidion attached to eupathidium. Chelicerae (length 164) with two barbed setae; *cha* (61) longer than *chb* (45). Trägårdh’s organ distinct.

*Epimeral and lateral podosomal regions*. Epimeral setal formula: 3–1–3–3. All setae setiform, slightly barbed; *1a*, *1b*, *1c*, *2a*, *3a*, *3b*, *4a*, *4b* (16–20) shorter than and *3c*, *4c* (65–69). Setae *1a*, *2a*, *3a* removed from each other. Pedotecta I, II, discidia and circumpedal carinae normally developed.

*Anogenital region*. Five pairs of genital (12–16), one pair of aggenital (*ag*, 16–20), three pairs of adanal (*ad*_1_, *ad*_2_, 24–32, *ad*_3_, 20) and two pairs of anal (16–20) setae setiform, indistinctly barbed. Lyrifissures *iad* located in paraanal position. Adanal setae *ad*_3_ inserted in lateral position, removed from the anal aperture.

*Legs*. Generally, similar to *Neoribates (Neoribates) parabulanovae* sp. n. (Table 1).

#### Material examined.

Three specimens (holotype: male; two paratypes: one male and one female) of *Neoribates (Neoribates) paramacrosacculatus* sp. n. are from eastern Nepal, 27°19'N, 87°78'E, 2770 m a.s.l., Panchthar District, upper course of Mai Majuwa river, pasture Dhorpar Kharka, soil in mixed broadleaved forest, soil, 27–28.VIII.1983, collected by J. Martens & B. Daams.

#### Type deposition.

The holotype and one paratype are deposited in the collection of the Senckenberg Institution Frankfurt, Germany; one paratype is deposited in the collection of the Tyumen State University Museum of Zoology, Tyumen, Russia.

#### Etymology.

The prefix *para* is Latin meaning “near” and refers to the similarity between the new species and the species *Neoribates (Neoribates) macrosacculatus* Aoki, 1966.

#### Remarks.

*Neoribates (Neoribates) paramacrosacculatus* sp. n. is morphologically similar to *Neoribates (Neoribates) macrosacculatus* Aoki, 1966 from the eastern Palaearctic region in having the long openings of notogastral sacculi. However, the new species differs from *Neoribates (Neoribates) macrosacculatus* by the larger body size (780–830 × 547–581 versus 630–760 × 430–480 in *Neoribates (Neoribates) macrosacculatus*), foveolate body integument and striate anal figs (versus smooth in *Neoribates (Neoribates) macrosacculatus*), bothridial setae of medium size, length of stalk not longer than head (versus long, stalk obviously longer than head in *Neoribates (Neoribates) macrosacculatus*), presence of five pairs of genital setae (versus four pairs in *Neoribates (Neoribates) macrosacculatus*) and absence of lamellar setae (versus present in *Neoribates (Neoribates) macrosacculatus*) and presence of very short interlamellar setae (versus long in *Neoribates (Neoribates) macrosacculatus*).

**Figures 5–6.**
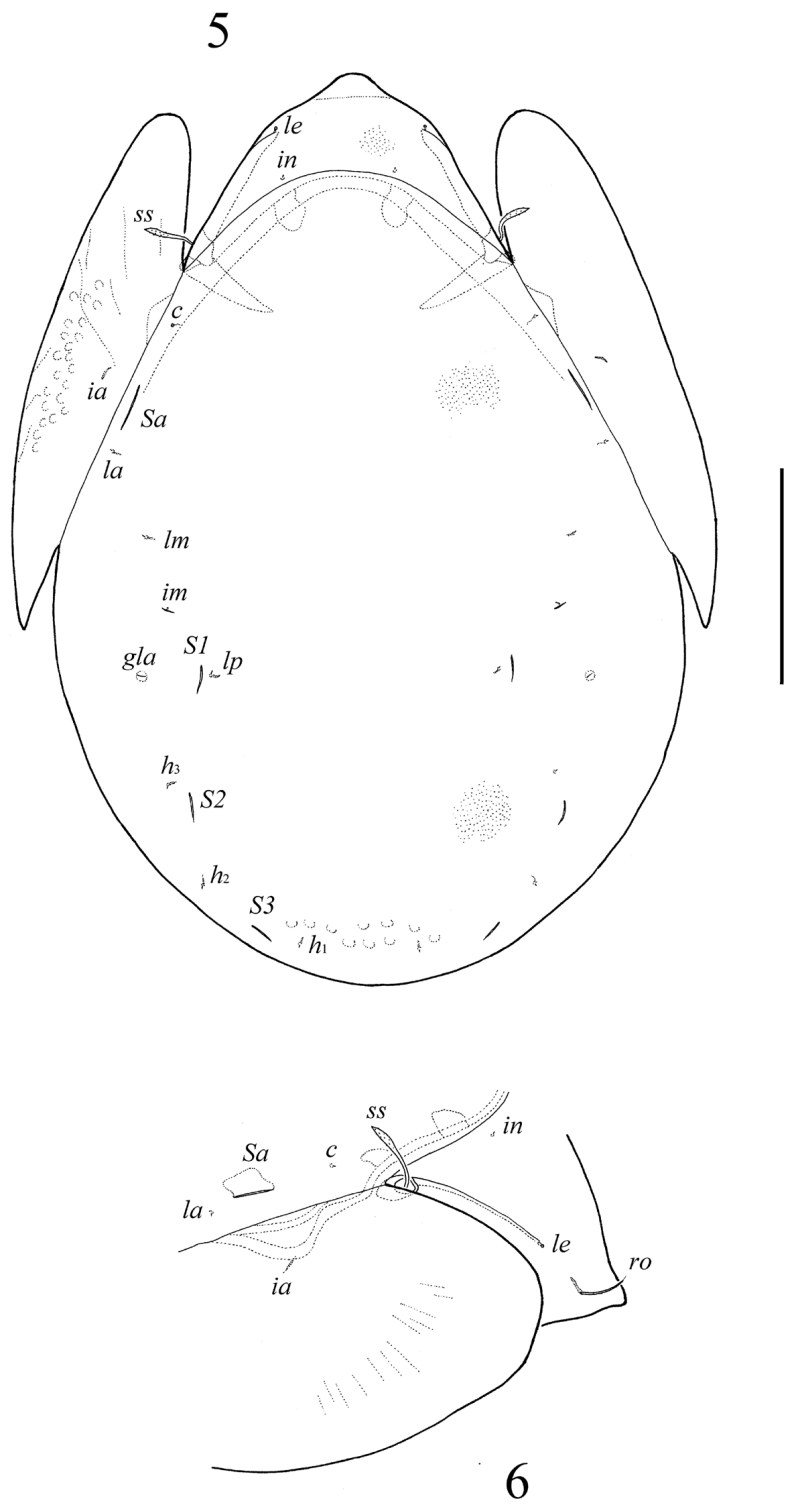
*Neoribates (Neoribates) paramacrosacculatus* sp. n., adult: **5** dorsal view **6** lateral view of prodorsum, anterior part of notogaster and pteromorph. Scale bar 200 μm.

**Figures 7–8.**
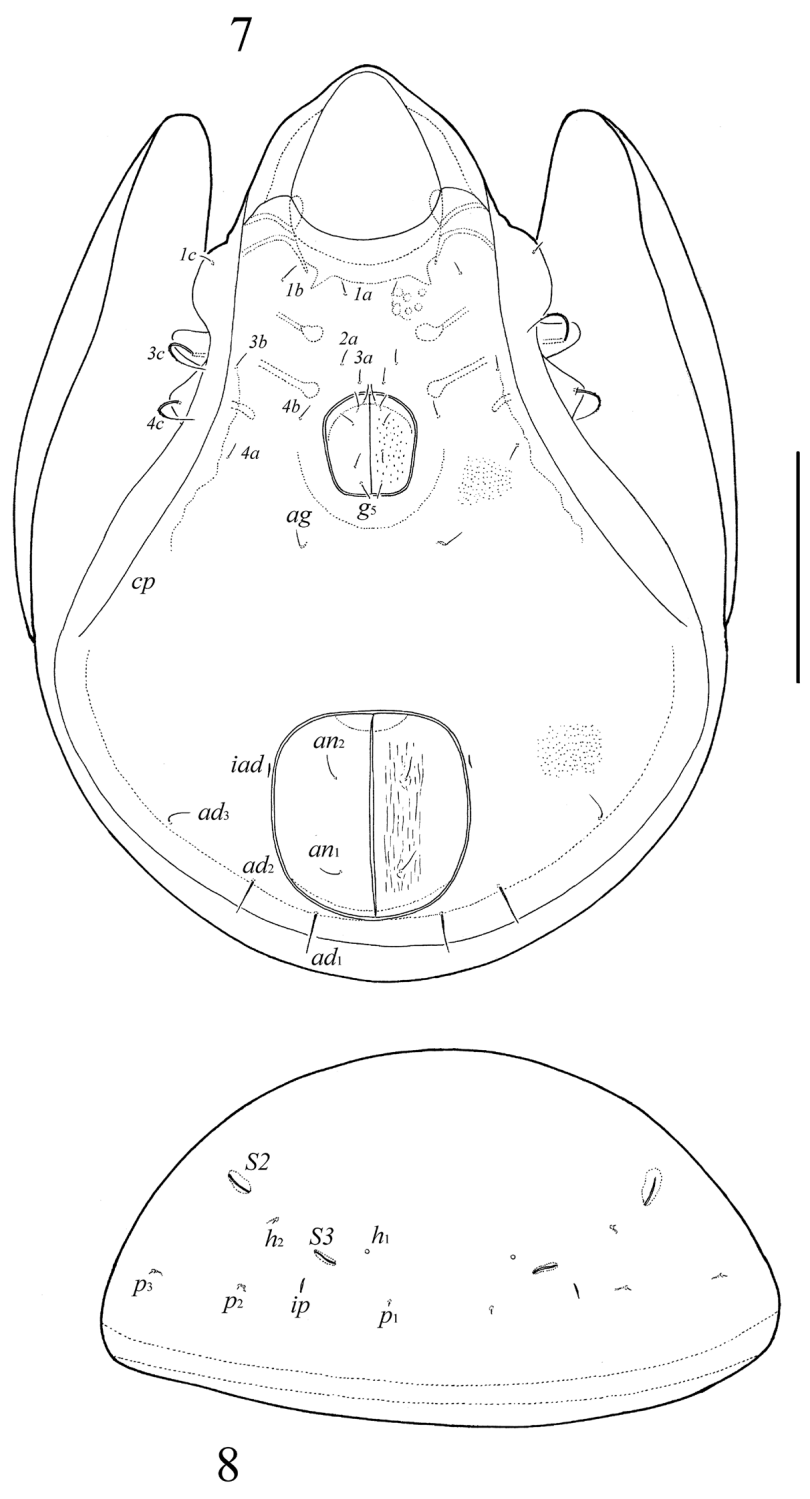
*Neoribates (Neoribates) paramacrosacculatus* sp. n., adult: **7** ventral view (gnathosoma and legs not illustrated) **8** posterior view of notogaster (pteromorphs not illustrated). Scale bar 200 μm.

### Neoribates (Neoribates) pararotundus sp. n.

Taxon classificationAnimaliaSarcoptiformes Parakalummidae

http://zoobank.org/DB039B08-2436-47C2-B0F6-5750796FC391

Figs 9 –12

#### Diagnosis.

Body size: 796–946 × 614–664. Body surface densely microfoveolate. Lamellae with distal tooth. Rostral and lamellar setae setiform, barbed. Interlamellar and bothridial setae setiform, with attenuate tip, smooth. Notogastral setae represented by 14 pairs of alveoli. Saccular openings short. Subcapitular setae *h* and *m* longer than *a*; *m* thinnest. Five pairs of genital setae present. Adanal setae *ad*_1_, *ad*_2_ of medium size, with attenuate tip. Adanal setae *ad*_3_ inserted in preanal position.

#### Description.

*Measurements*. Body length: 946 (holotype), 796, 929 (two paratypes); notogaster width: 664 (holotype), 614, 647 (two paratypes).

*Integument*. Body color dark brown. Body surface densely microfoveolate (diameter of microfoveoles less than 1; visible only under high magnification in dissected specimens, × 1000). Numerous stria located around the genital and anal apertures, poorly visible.

*Prodorsum*. Rostrum weakly protruding, rounded. Lamellae distinct, reaching the insertions of alveoli of lamellar setae, with distal tooth (*t*). Rostral (80–98) and lamellar (131–151) setae setiform, barbed. Interlamellar (274–307) and bothridial (155–172) setae setiform, with attenuate tip, smooth. Exobothridial setae short (*ex*, 12), thin, smooth.

*Notogaster*. Anterior notogastral margin convex. Notogastral setae represented by 14 pairs of alveoli, one pair of *c*-row absent. Four pairs of sacculi present, their openings short, thin. Alveoli *lp* inserted posteriorly to *S1*. Opisthonotal gland openings located laterally to succuli *S1*. Lyrifissures *im* located between *gla* and setal alveolus *lm.*

*Gnathosoma*. Generally, morphology of subcapitulum, palps and chelicerae typical as for most *Neoribates (Neoribates)* (see for example Grishina and Vladimirova 2009; Ermilov and Kalúz 2013; Ermilov and Anichkin 2014). Subcapitulum longer than wide (205 × 147). Subcapitular setae setiform, slightly barbed; *a* (24–28) and *h* (32–36) thicker than *m* (32–36). Two pairs of adoral setae (16–20) setiform, densely bilaterally barbed. Palps (length 110) with setation 0–2–1–3–9(+ω). Solenidion attached to eupathidium. Chelicerae (length 221) with two barbed setae; *cha* (57) longer than *chb* (36). Trägårdh’s organ distinct.

*Epimeral and lateral podosomal regions*. Epimeral setal formula: 3–1–3–3. All setae setiform, slightly barbed; *1a*, *1b*, *1c*, *2a*, *3a*, *3b*, *4a*, *4b* (32–41) shorter than and *3c*, *4c* (65–73). Setae *1a*, *2a*, *3a* located close to each other. Pedotecta I, II, discidia and circumpedal carinae normally developed.

*Anogenital region*. Five pairs of genital (20–24), one pair of aggenital (36–45), three pairs of adanal (*ad*_1_, 82–90, *ad*_2_, 57–61, *ad*_3_, 45–53) and two pairs of anal (36–45) setae setiform (except *ad*_1_, *ad*_2_ with attenuate tip), slightly barbed. Lyrifissures *iad* located in paraanal position, diagonally to the anal aperture. Adanal setae *ad*_3_ inserted in preanal position.

*Legs*. Generally, similar to *Neoribates (Neoribates) parabulanovae* sp. n. (Table 1).

#### Material examined.

Three specimens (holotype: male; two paratypes: one male and one female) of *Neoribates (Neoribates) pararotundus* sp. n. are from central Nepal, 27°96' N, 84°97' E, 1100–1300 m a.s.l., Gorkha District, Buri Gandaki valley, riverine forest, between Dobhan and Jagat, soil, 13.VIII.1983, collected by J. Martens & W. Schawaller.

#### Type deposition.

The holotype and one paratype are deposited in the collection of the Senckenberg Institution Frankfurt, Germany; one paratype is deposited in the collection of the Tyumen State University Museum of Zoology, Tyumen, Russia.

#### Etymology.

The prefix *para* is Latin meaning “near” and refers to the similarity between the new species and the species *Neoribates (Neoribates) rotundus* Aoki, 1982.

#### Remarks.

*Neoribates (Neoribates) pararotundus* sp. n. is morphologically similar to *Neoribates (Neoribates) rotundus* Aoki, 1982 from Japan and Taiwan in having the large body size and setiform, smooth bothridial setae. However, the new species differs from *Neoribates (Neoribates) rotundus* by the presence of 14 pairs of notogastral setal alveoli (versus 10 pairs in *Neoribates (Neoribates) rotundus*), microfoveolate body integument (versus smooth in *Neoribates (Neoribates) rotundus*), lamellae with distal tooth (versus without tooth in *Neoribates (Neoribates) rotundus*) and interlamellar setae longer than bothridial setae (versus shorter in *Neoribates (Neoribates) rotundus*).

**Figures 9–10.**
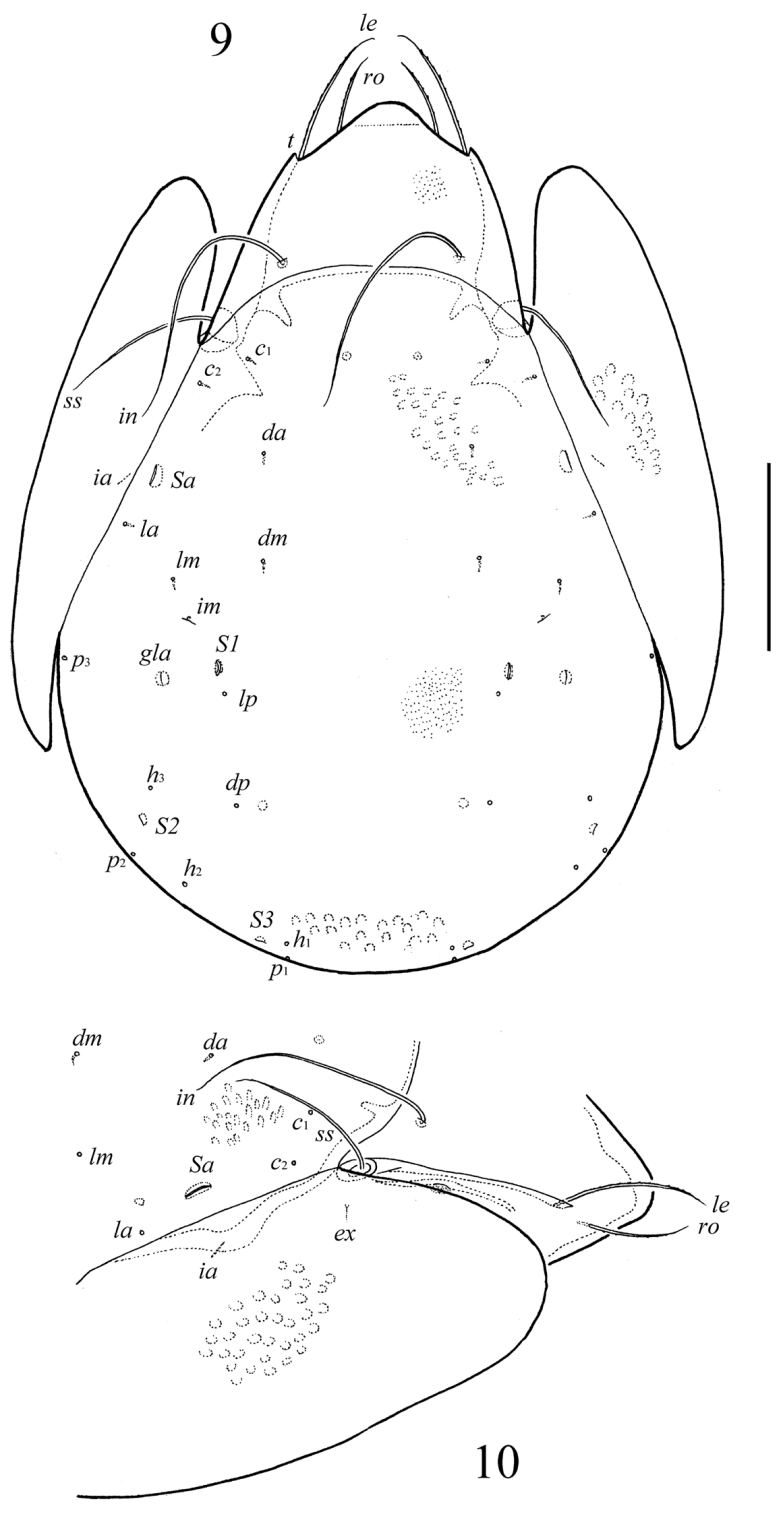
*Neoribates (Neoribates) pararotundus* sp. n., adult: **9** dorsal view **10** lateral view of prodorsum, anterior part of notogaster and pteromorph. Scale bar 200 μm.

**Figures 11–12.**
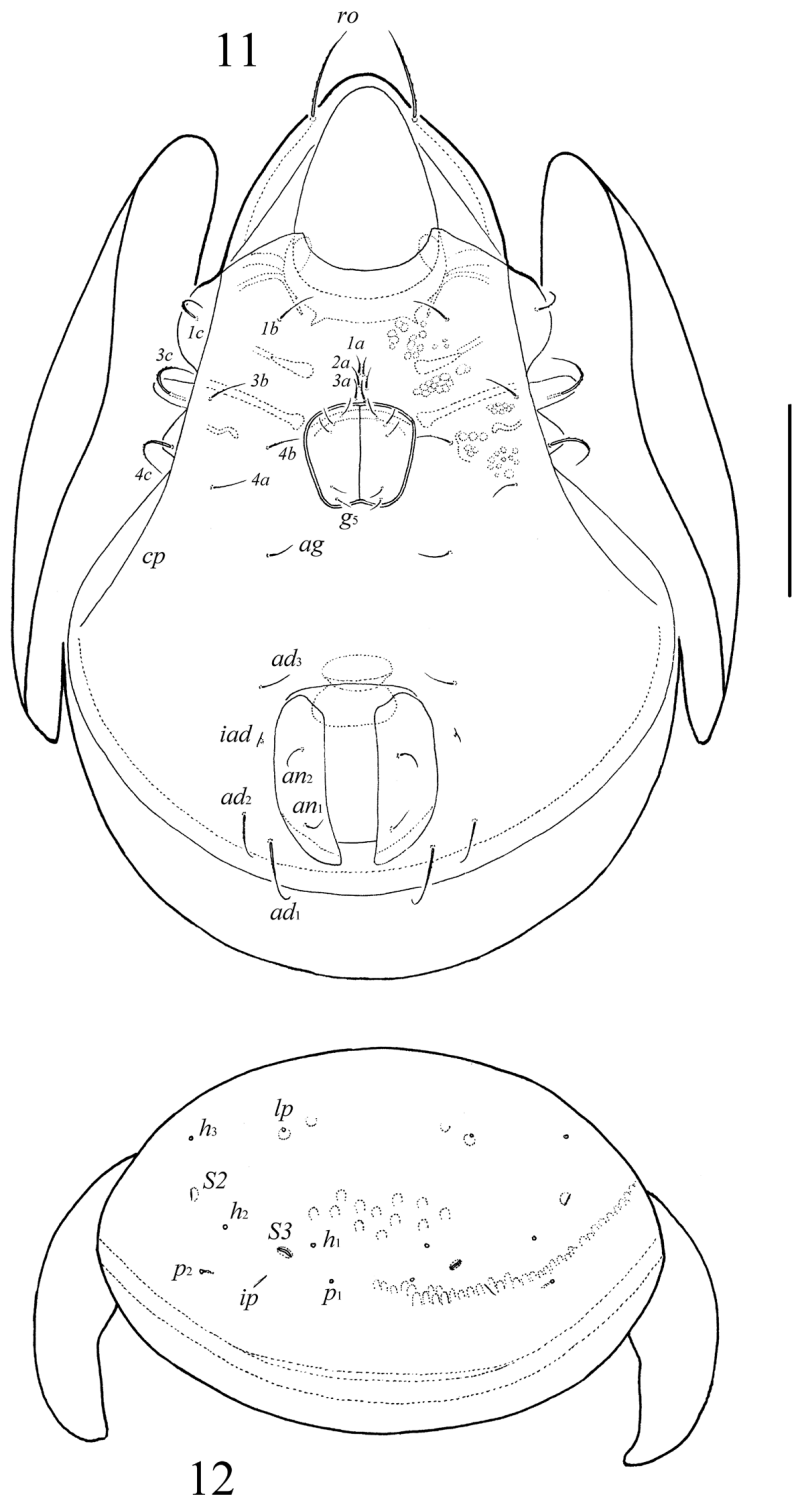
*Neoribates (Neoribates) pararotundus* sp. n., adult: **11** ventral view (gnathosoma and legs not illustrated) **12** posterior view of notogaster. Scale bar 200 μm.

## Supplementary Material

XML Treatment for Neoribates (Neoribates) parabulanovae

XML Treatment for Neoribates (Neoribates) paramacrosacculatus

XML Treatment for Neoribates (Neoribates) pararotundus

## Footnotes

1 Results of the Himalaya Expeditions (1980, 1983) of Dr. Jochen Martens.
